# Supplementary material for: Targeting cyclin-dependent kinases for the treatment of pulmonary arterial hypertension
Source: Nat Commun. 2019 May 17;10:2204. doi: 10.1038/s41467-019-10135-x (PMC6525202; doi:10.1038/s41467-019-10135-x)
Supplement: Supplementary file 1 — Supplementary information [file 41467_2019_10135_MOESM1_ESM.pdf]

Supplementary information

Targeting cyclin-dependent kinases for the treatment of pulmonary arterial hypertension

Weiss and Neubauer et al.

Supplementary figures 1 to 12

Supplementary table 1

Supplementary discussion

Supplementary methods

Supplementary Figure 1

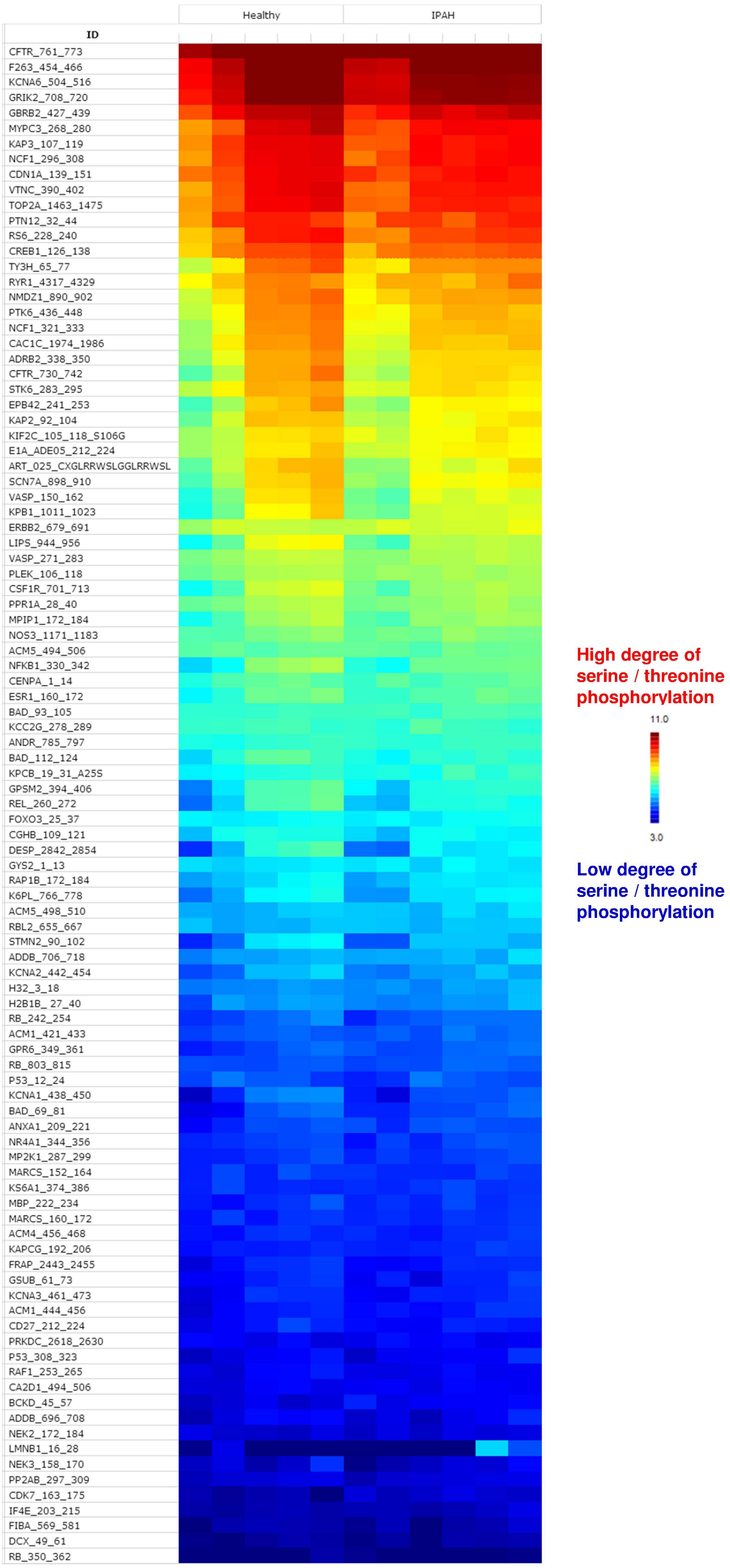

Supplementary Figure 1: Heat map from the peptide micro array obtained from all individual samples. Raw data for all individual HPASMC samples from both entities (healthy n=5; IPAH n=6) presented as a heat map with log-transformed fluorescence signals due to kinase-mediated substrate serine/threonine phosphorylation. Based on those signal intensities an upstream kinase activity was predicted with increased levels for distinct CDKs. Source data are provided as a Source Data file.

Supplementary Figure 2

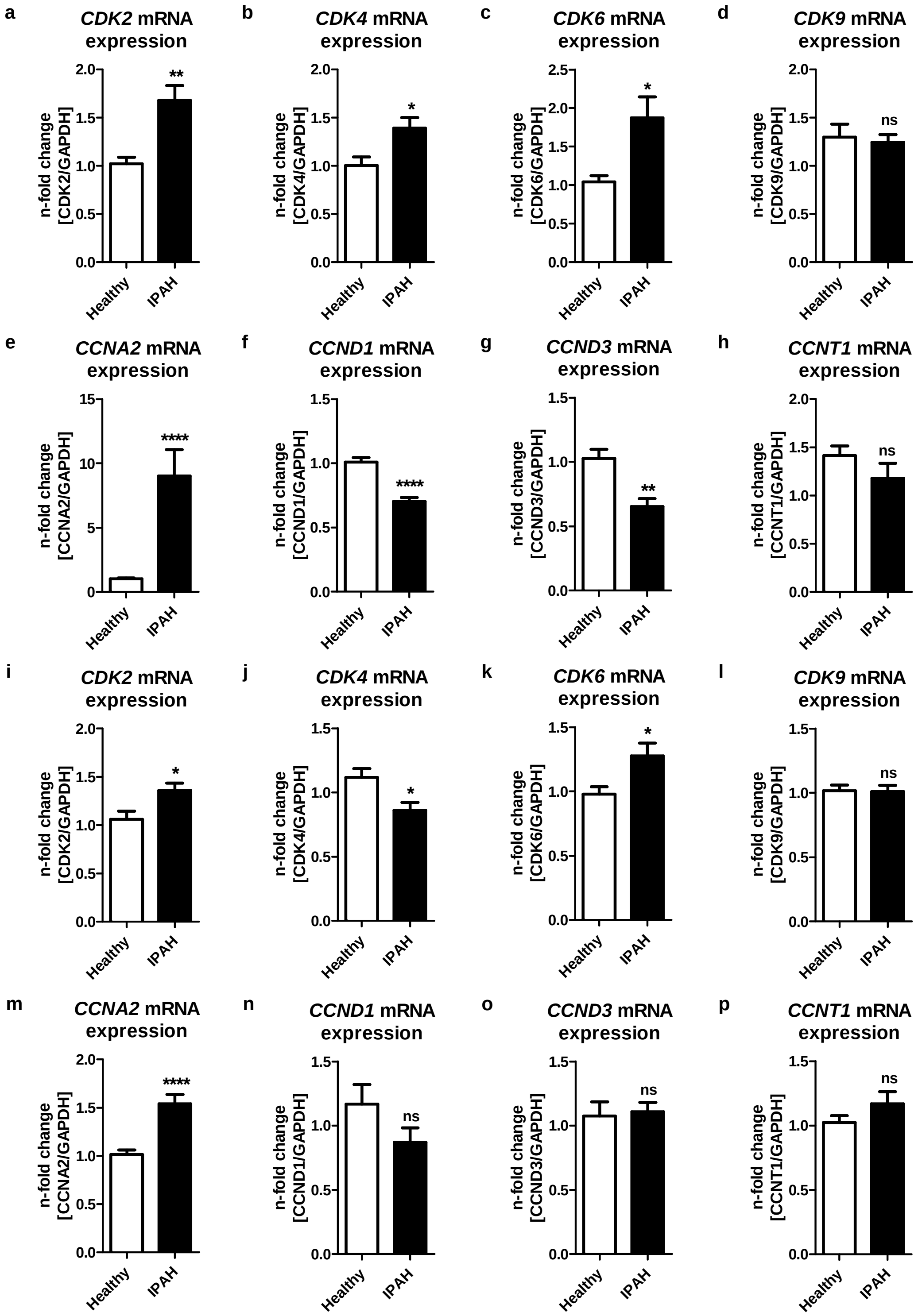

Supplementary Figure 2: Analysis of *CDK* and *cyclin* mRNA expression in human specimens. mRNA analysis normalized to *GAPDH* as reference gene for different *CDKs* (**a-d**, **i-l**) and *cyclins* (**e-h**, **m-p**) in HPASMCs from healthy individuals and IPAH-patients (n=2-4, each) (**a-h**) and in homogenates (**i-p**) of freshly isolated and processed lung biopsies from healthy individuals (n=3) and IPAH patients (n=5). All data are presented as mean±SEM of the n-fold change ($2^{-\Delta\Delta Ct}$) compared with a healthy control and analyzed statistically using a Mann-Whitney test; * p < 0.05, ** p < 0.01, **** p < 0.0001. Source data are provided as a Source Data file.

Supplementary Figure 3

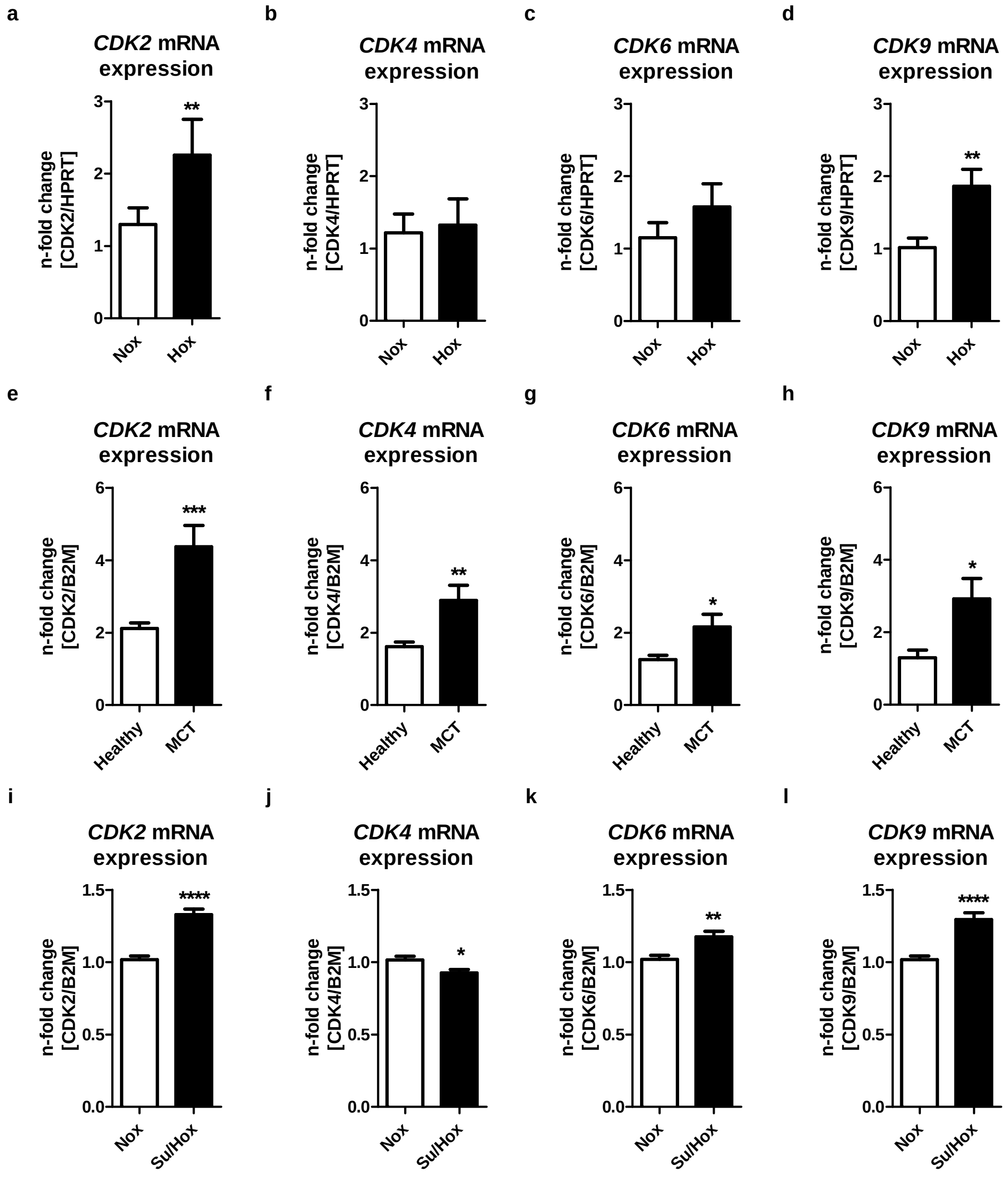

Supplementary Figure 3: Analysis of *CDK* mRNA expression in the experimental models of PAH. (**a-d**) Male C57BL/6J wild-type mice (age 12-18 weeks, mean body weight of 20 g) were maintained under normoxic conditions (n=3-6) or in hypoxic chambers with reduced O_2 saturation for three weeks (n=3-5). *CDK2*, *CDK4*, *CDK6* and *CDK9* mRNA expression in murine lung homogenates was normalized to *hypoxanthine guanine phosphoribosyl transferase* (*HPRT*) as a reference gene. (**e-h**) Healthy male Sprague-Dawley rats (age 12 weeks, mean body weight of 300-350 g, n=5) were compared with diseased rats five weeks after MCT injection (MCT, n=5). (**i-l**) Healthy male Wistar-Kyoto rats (age 12 weeks, mean body weight of 300-350 g, n=8) kept under normoxic conditions were compared with diseased rats which underwent a three weeks housing period with hypoxic exposure after an initial Sugen (Su5416) injection followed by two weeks of re-exposure to normoxic conditions (Su/Hox, n=8). *CDK2*, *CDK4*, *CDK6* and *CDK9* mRNA expression in rat lung homogenates was normalized to *B2M* as a reference gene. All data are presented as mean±SEM of the n-fold change ($2^{-\Delta\Delta Ct}$) compared with a healthy control. Statistical analysis was performed using an unpaired Student´s t-test; * $p < 0.05$, ** $p < 0.01$, *** $p < 0.001$, **** $p < 0.0001$. Source data are provided as a Source Data file.

Supplementary Figure 4

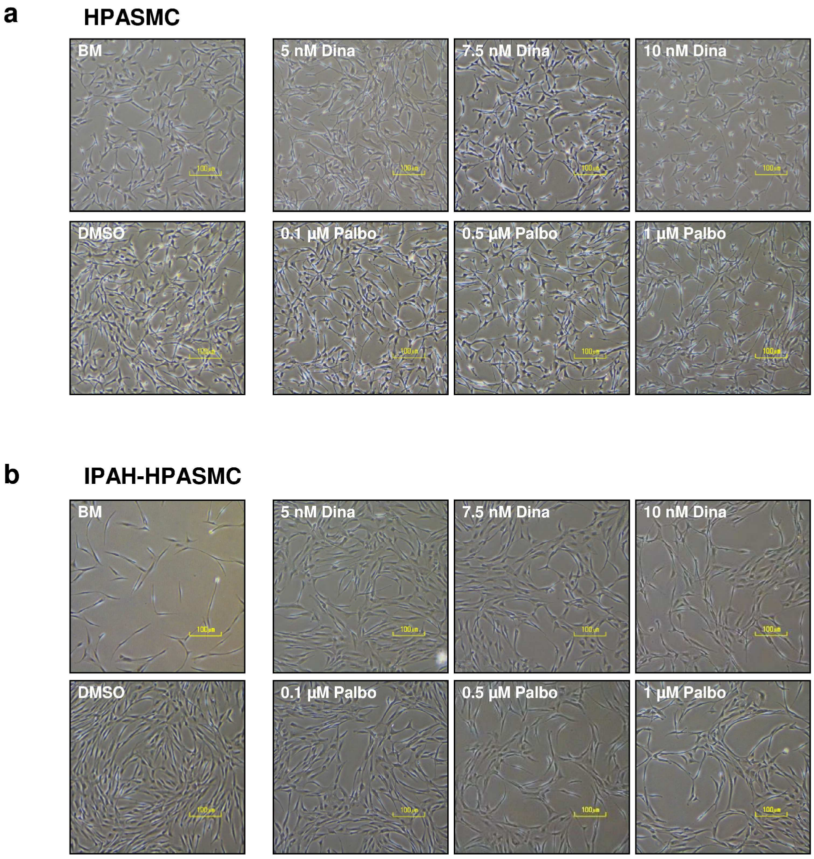

Supplementary Figure 4: Microscopic analysis of healthy and diseased IPAH-HPASMCs. Cells were synchronized in basal media (BM) and then treated with the indicated concentrations of dinaciclib or palbociclib in the presence of growth media (GM-2) for 24 h. Representative microscopic images (400-fold magnification with a scale bar of 100 µm) for HPASMCs (**a**) and IPAH-HPASMCs (**b**) were taken to monitor cell density and morphology before further analysis (**Figure 5**).

Supplementary Figure 5

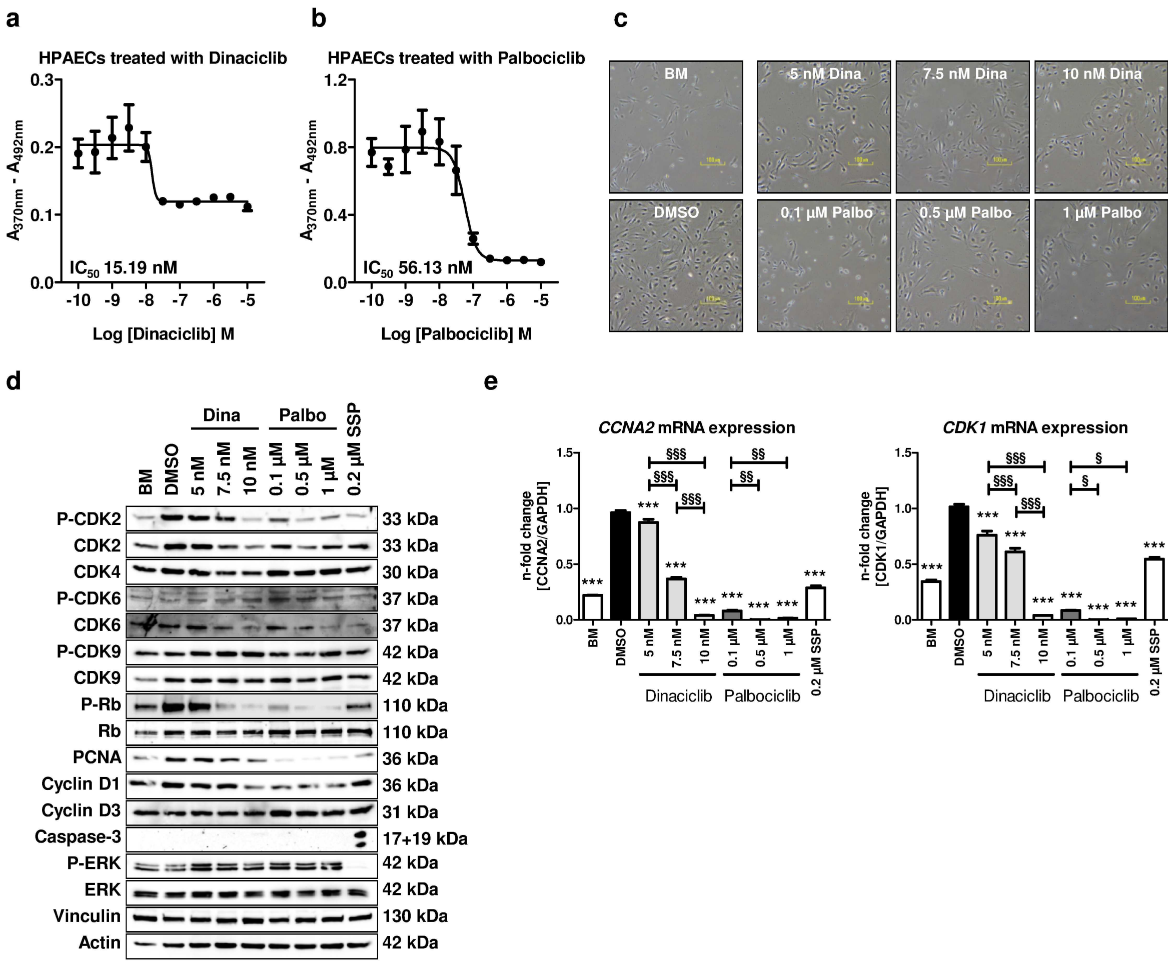

Supplementary Figure 5: Evaluation of IC_{50} concentrations of the CDK inhibitors dinaciclib and palbociclib on proliferation, and their effects in CDK-Rb-E2F signaling of healthy HPAECs. Cells were synchronized in endothelial specific basal media (ECBM supplemented with 0.5% FCS) and then treated with the indicated concentrations of dinaciclib or palbociclib or DMSO as control in the presence of growth media (ECGM) for 24 h. IC_{50} graphs determining the half maximal inhibitory concentration of dinaciclib (**a**) and palbociclib (**b**) to block ECGM induced proliferation of healthy HPAECs as determined by BrdU incorporation (relative absorbance [A_{370nm}-A_{492nm}]). IC_{50} values were calculated from measurements of one primary cell isolate (run in two independently performed triplicates) by the non-linear regression (curve fit) module with a variable slope using four parameters as provided by GraphPad Prism software. Representative microscopic images from distinct cell culture conditions (**c**) were taken with a 400-fold magnification (with a scale bar of 100 µm) prior the performance of representative Western blots (**d**) for the detailed analyses of cellular signaling on protein level after 24 hours of treatment with dinaciclib, palbociclib, or staurosporine (SSP) in healthy HPAECs with regard to CDK activation and downstream Rb-E2F pathway induction. *CCNA2* (left) and *CDK1* (right) mRNA expression (normalized to *GAPDH* as a house keeping gene) of healthy HPAECs (**e**) upon 24 hours of inhibitor exposure. All data from one primary cell isolate (run twice in triplicates each) are presented as mean±SEM of the n-fold change ($2^{-\Delta\Delta Ct}$) compared with DMSO treated control samples (black bar). Statistical analysis was performed using one-way ANOVA with Newman-Keuls post-hoc test for multiple comparisons; *** $p < 0.001$. P-values for distinct conditions are only given for their comparison with DMSO-treated control cells (black bar), and the significance of the difference between the three conditions with various concentrations of each of the CDK inhibitors is represented as follows: § $p < 0.05$, §§ $p < 0.01$, §§§ $p < 0.001$. Source data are provided as a Source Data file.

Supplementary Figure 6

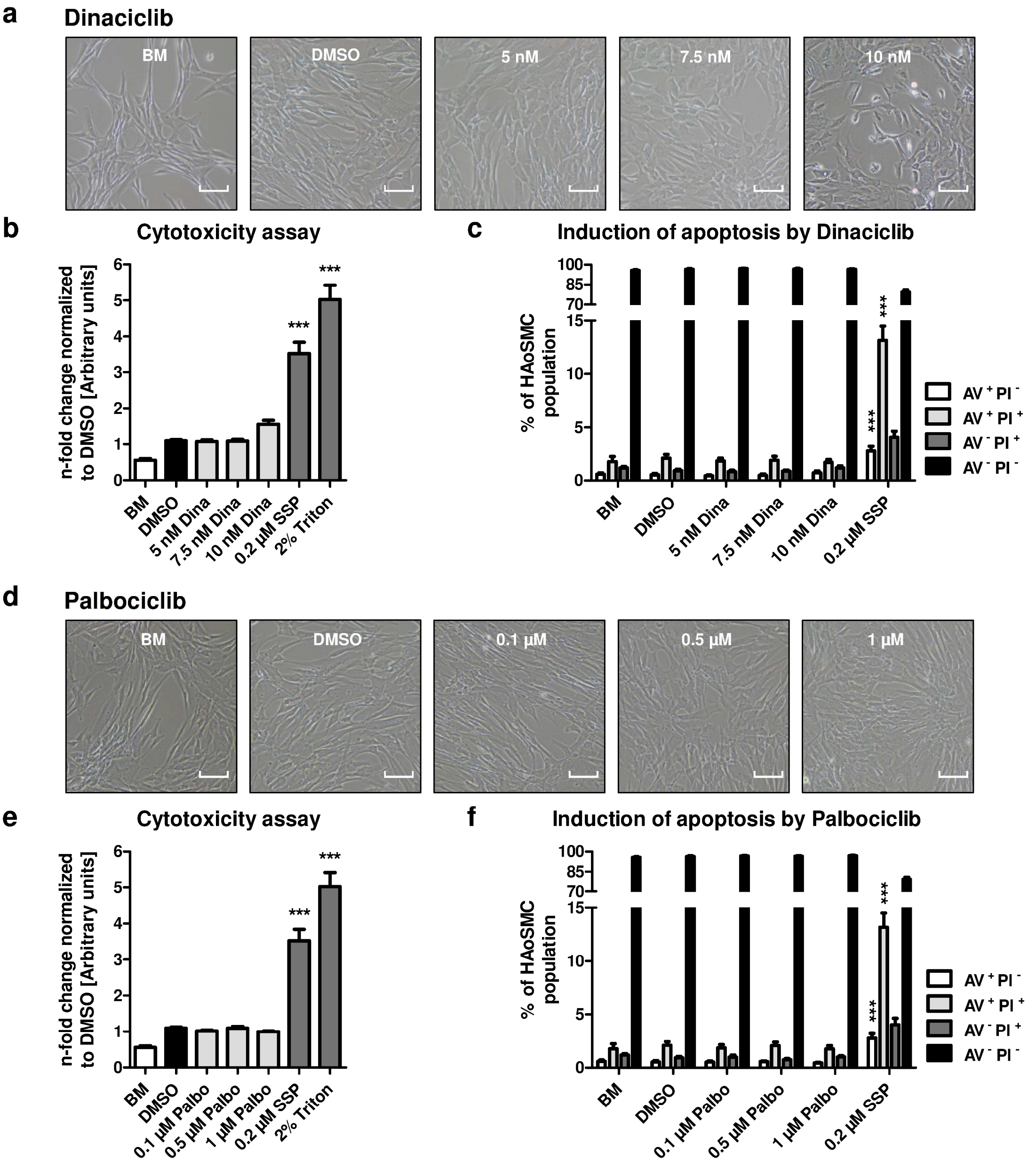

Supplementary Figure 6: Effects of dinaciclib and palbociclib on survival and apoptosis of human aortic smooth muscle cells (HAoSMCs). HAoSMCs were starved and then induced for 24 h with growth media with the indicated concentrations of dinaciclib (upper panels) and palbociclib (lower panels) or DMSO as control. (**a**, **d**) Representative images at 400-fold magnification (with a scale bar of 100 µm) were taken to monitor cell density and morphology. (**b**, **e**) Cytotoxicity was investigated by LDH assay. Data from three independent experiments performed in triplicates are presented as mean±SEM of the n-fold change normalized to a DMSO-treated control sample. Statistical analysis was performed using one-way ANOVA with Newman-Keuls post-hoc test for multiple comparisons; *** $p < 0.001$. P-values for distinct conditions are only given for their comparison with DMSO treated-control cells (black bar). (**c, f**) Combined staining of cells with AV and PI was detected by flow cytometry to reveal early apoptotic (AV^+ PI^-, white) and late apoptotic (AV^+ PI^+, light-grey), dead (AV^- PI^+, dark-grey), and living cells (AV^- PI^-, black). Data from three independent experiments performed in triplicates are presented as mean±SEM of the percentage of indicated populations. Statistical analysis was performed using one-way ANOVA with Dunnett´s post-hoc test to compare all bars versus DMSO control; *** $p < 0.001$. Source data are provided as a Source Data file.

Supplementary Figure 7

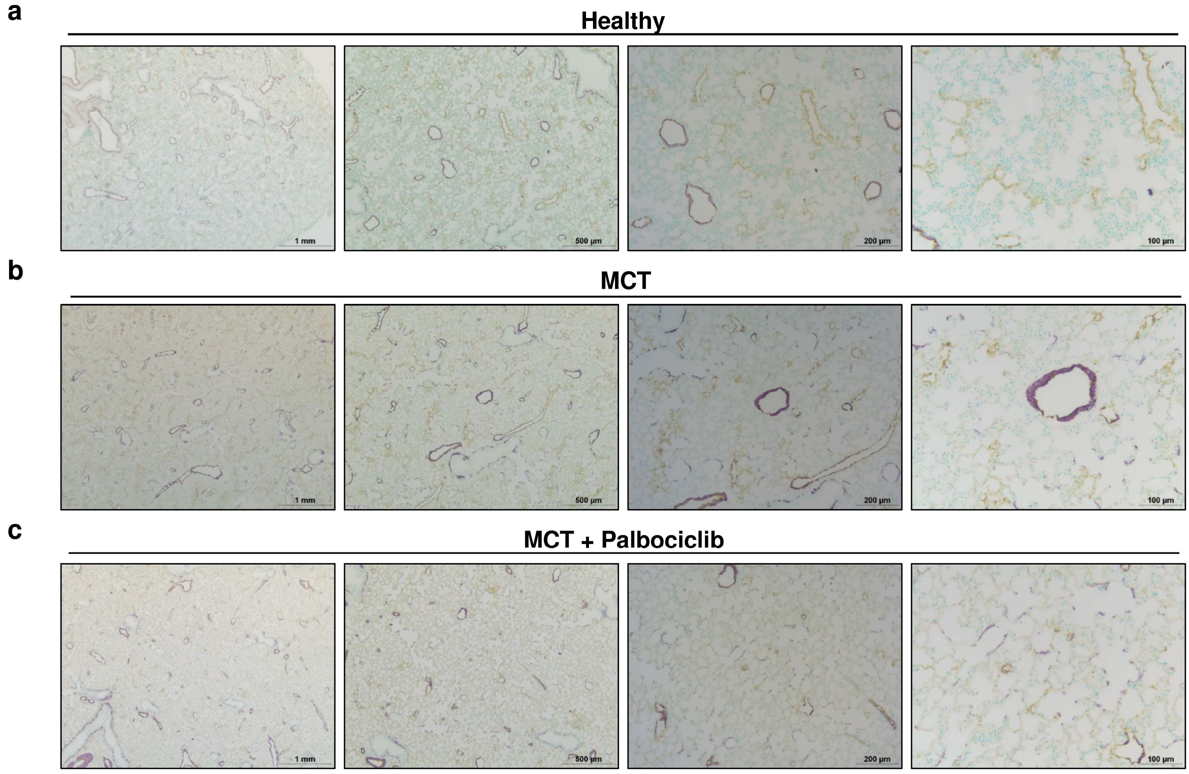

Supplementary Figure 7: Structural remodeling at the level of individual pulmonary arteries for the MCT rat model of PAH models. Representative images from microscopic fields of interest with multiple vessels for lungs explanted from healthy (**a**), diseased (**b**) and palbociclib treated (**c**) rats stained for α-SMA and vWF. Magnifications from left to right: 25-fold, 50-fold, 100-fold, and 200-fold.

Supplementary Figure 8

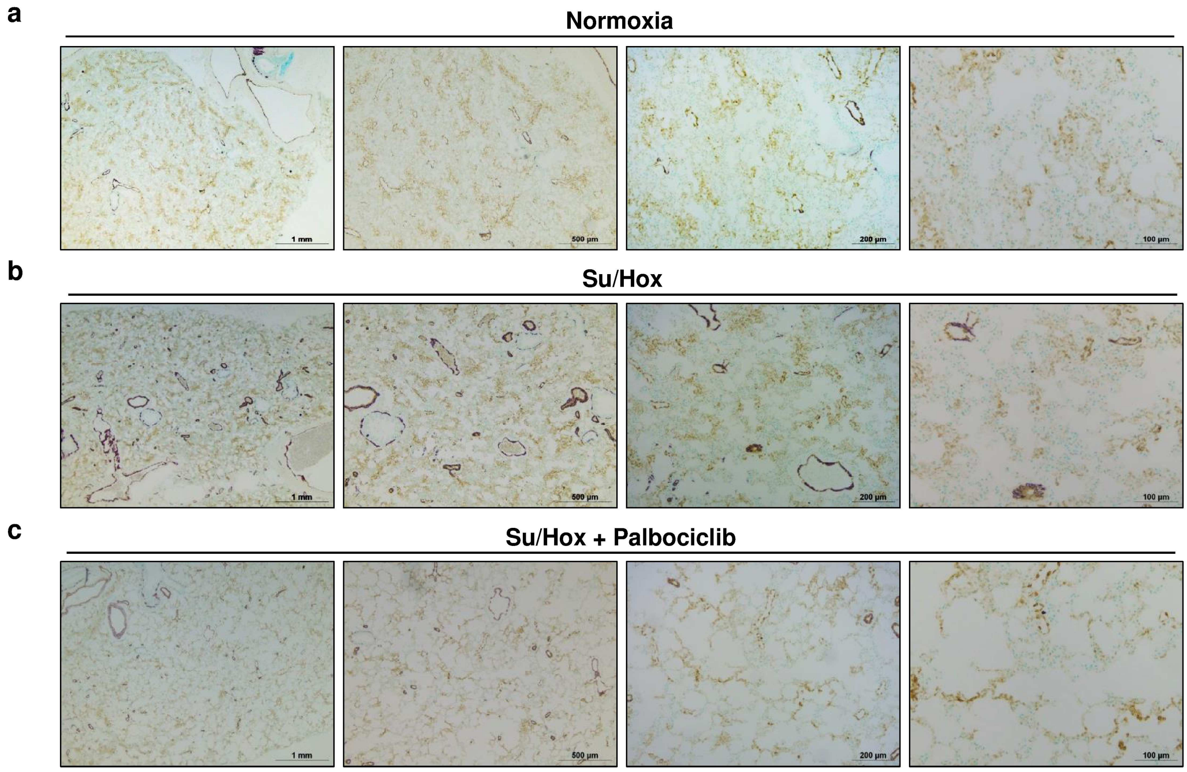

Supplementary Figure 8: Structural remodeling at the level of individual pulmonary arteries for the Su/Hox rat model of PAH models. Representative images from microscopic fields of interest with multiple vessels for lungs explanted from the normoxic (**a**), diseased (**b**) and palbociclib treated (**c**) rats stained for α-SMA and vWF. Magnifications from left to right: 25-fold, 50-fold, 100-fold, and 200-fold.

Supplementary Figure 9

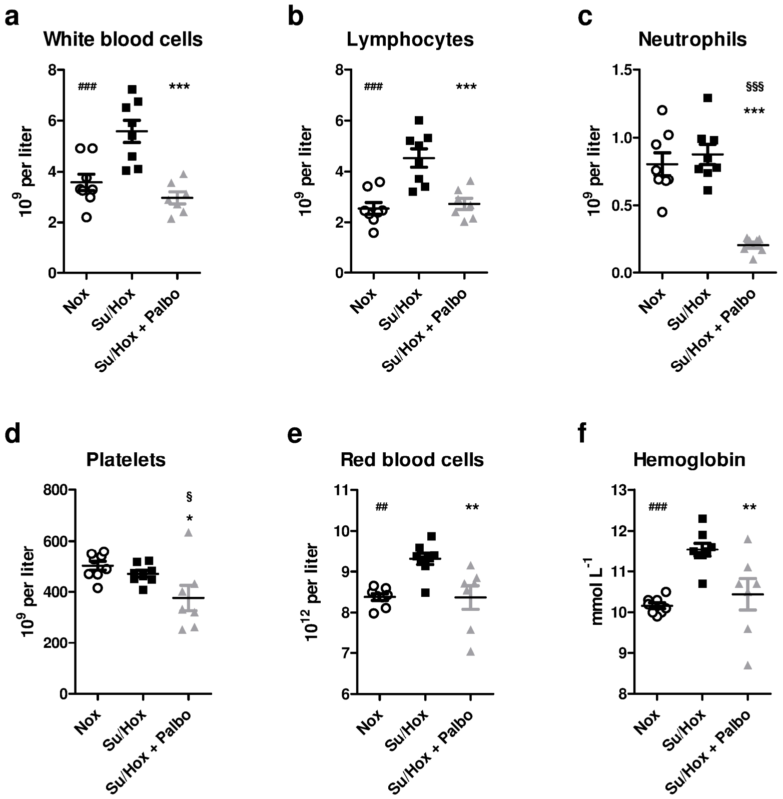

Supplementary Figure 9: Blood analysis of laboratory rats. Blood analysis from samples derived from all rats of the Su/Hox study (Nox n=8; Su/Hox n=8; Su/Hox+Palbo n=7). Distinct parameters indicating any possible negative side effects of palbociclib based on the known toxicology profile were analyzed by the flow cytometry. * $p < 0.05$; ** $p < 0.01$, *** $p < 0.001$ for Su/Hox+Palbo versus Su/Hox; § $p < 0.05$; §§§ $p < 0.001$ for Su/Hox+Palbo versus Nox; $^{\#\#}$ $p < 0.01$, $^{\#\#\#}$ $p < 0.001$ for Nox versus Su/Hox. Source data are provided as a Source Data file.

Supplementary Figure 10

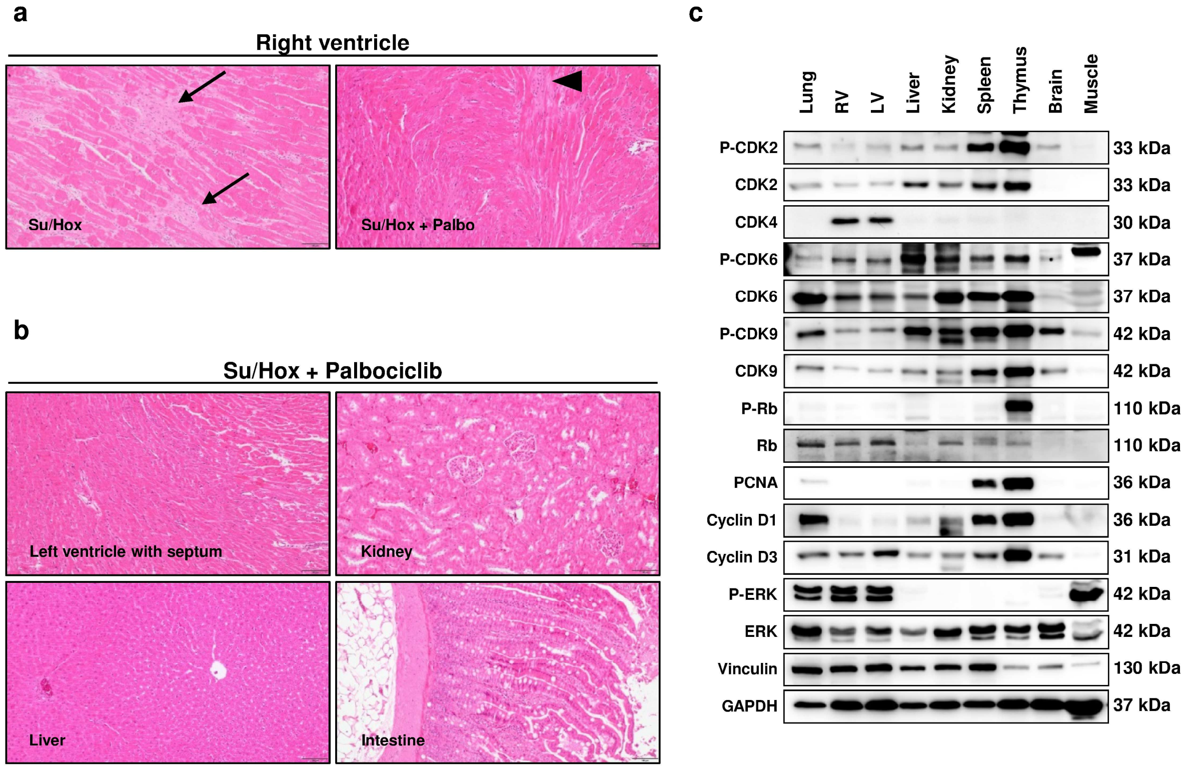

Supplementary Figure 10: Evaluation of distinct organs from the experimental PAH models. Representative histopathological images of the right ventricle (**a**) and other organs (**b**) of H&E stained FFPE sections from the Su/Hox rat model. Images were taken at adequate magnification with a scale bar of 100 µm. (**c**) Representative Western blot of several organs of healthy rats (n=5) with regard to (P-)CDK expression and activation of the downstream Rb-E2F signaling pathway as well as for (P-)ERK. RV: right ventricle, LV: left ventricle. Expression of glyceraldehyde-3-phosphate dehydrogenase (GAPDH) protein is shown as a loading control. Source data are provided as a Source Data file.

Supplementary Figure 11

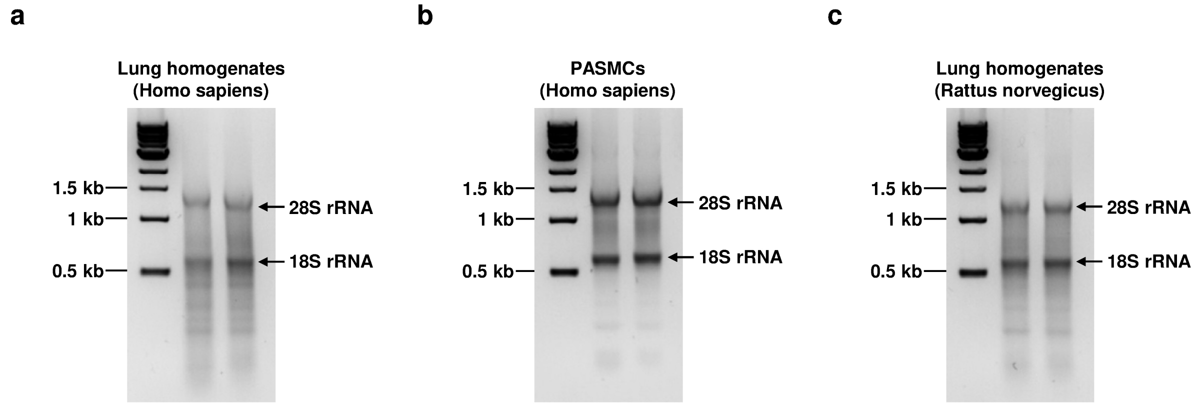

Supplementary Figure 11: Agarose gel demonstrating the quality and integrity of isolated RNA. 1 µg RNA from two representative samples for the three different specimens were analyzed on a denaturing agarose gel (1.5 %) containing 2.2 M formaldehyde stained with ethidium bromide according to standard procedures (Maniatis et al., 2001, 3th edition, Molecular cloning: A laboratory manual, Cold Spring Harbor Laboratory, New York). The presence of 28S and 18S rRNA bands indicates proper purification techniques prior to subsequent cDNA synthesis and quantitative real-time PCR analysis.

Supplementary Figure 12

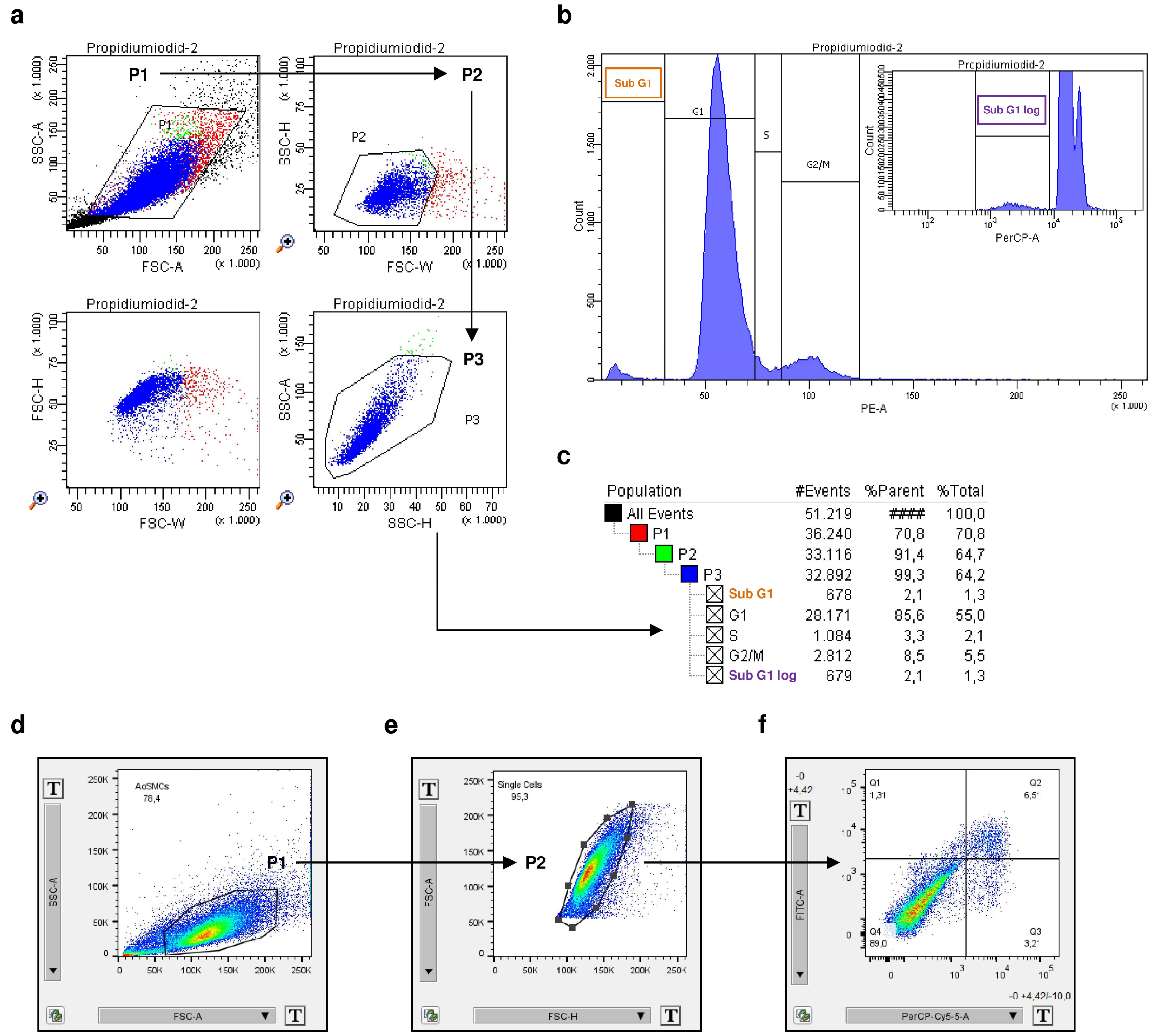

Supplementary Figure 12: Gating strategy for cell cycle analysis and apoptosis detection. Samples were prepared as mentioned in the method section and subsequently analyzed for cell cycle distribution (**a**-**c**) or for induction of apoptosis (**d**-**f**). Cells were first assessed in the FSC-A/SSC-A dot plot to exclude cell debris (*P1*) and doublet discrimination was carried out by additional plots to remove doublets and cell clumps (*P2* and *P3*) (**a**). Finally, fragmented DNA (low signals) as well as the different cell cycle phases (increasing signals) were recorded (**b**). Linear (orange, PE-A) and logarithmic (purple, PerCP-A) scaling of the x-axis led to identical event counts for the SubG1 population (**c**). For apoptosis detection, samples were analysed by FSC-A/SSC-A dot plot and cell debris was removed (*P1*) (**d**). Next, doublets were excluded (*P2*) (**e**) and finally, fluorescence was detected in the FITC (AV) and PerCP-Cy5.5 (PI) channel (**f**). A detailed description of the experimental design can be found in the main manuscript.

Supplementary Table 1

Gene	Species	NCBI reference	Forward primer: 5´-3´	Reverse primer: 5´-3´
CDK2	*Homo sapiens*	NM_001290230.1<br>NM_001798.4<br>NM_052827.3	CTCTGCTCTCACTGGCATTC	AGGTTTAAGGTCTCGGTGGAG
CDK2	*Mus musculus*	NM_016756.4<br>NM_183417.3	CACCCTAATATCGTCAAGCTGC	ATAAGCAGGTTCTGGGGCTT
CDK2	*Rattus norvegicus*	NM_199501.1	CTTTGGAGTCCCTGTCCGTA	GGGTCACCATTTCGGCAAAG
CDK4	*Homo sapiens*	NM_000075.3	ATGTGGAGTGTTGGCTGTATC	GGTTAAAAGTCAGCATTTCCAGC
CDK4	*Mus musculus*	NM_001355005.1<br>NM_009870.4	TAGCCGAGCGTAAGATCCC	CTTAACAAGGCCACCTCACG
CDK4	*Rattus norvegicus*	NM_053593.2	CCAGGACCTACGGACATACC	ACCAGAGCGTAACAACCACA
CDK6	*Homo sapiens*	NM_001145306.1<br>NM_001259.7	GTGGTCAGGTTGTTTGATGTGT	CGATGCACTACTCGGTGTGA
CDK6	*Mus musculus*	NM_009873.3	CGTGGTCAGGTTGTTTGATGT	TGCGGTTTCAGATCACGATG
CDK6	*Rattus norvegicus*	NM_001191861.1	AATCTTGGACGTCATCGGACT	CAGGTCTTGGAAGTACGGGT
CDK9	*Homo sapiens*	NM_001261.3	ATACGAGAAGCTCGCCAAGA	TAGGGGGAAGCTTTGGTTCG
CDK9	*Mus musculus*	NM_130860.3	TGCGATGAGGTCACCAAGTA	CGGTTATACGGTGAGGCTTTG
CDK9	*Rattus norvegicus*	NM_001007743.1	AAGGCACATTCGGGGAAGTA	TGCAGCGGTTATAGGGTGAA
CCNA2	*Homo sapiens*	NM_001237.4	ACCCAGAAAACCATTGGTCC	CATTTAACCTCCATTTCCCTAAGGT
CCNA2	*Rattus norvegicus*	NM_053702.3	CTCTTTACCCGGAGCCAGAA	CATTTAACCTCCATTTCCCTAAGGT
CDK1	*Homo sapiens*	NM_001170406.1<br>NM_001170407.1<br>NM_001320918.1<br>NM_001786.4<br>NM_033379.4	AATAAGCCGGGATCTACCATAC	CATGGCTACCACTTGACCTG
CDK1	*Rattus norvegicus*	NM_019296.1	GAACAGAGAGGGTCCGTTGTA	AGATTTCCCGGATTGCCGTA
CCND1	*Homo sapiens*	NM_053056.2	ACAGATCATCCGCAAACACG	GAGGCAGTCCGGGTCAC
CCND3	*Homo sapiens*	NM_001136017.3<br>NM_001136125.2<br>NM_001136126.2<br>NM_001287427.1<br>NM_001287434.1<br>NM_001760.4	AGGGATCACTGGCACTGAAG	GGCTGTGACATCTGTAGGAGT
CCNT1	*Homo sapiens*	NM_001240.3<br>NM_001277842.1	TGGAAAATAGCCCATCCCGT	GCTGGAGCCACAGAATTTCC
GAPDH	*Homo sapiens*	NM_001357943.1<br>NM_001357944.1<br>NM_002046.6	TTTTGCGTCGCCAGCCGAG	TGACCAGGCGCCCAATACGA
HPRT	*Mus musculus*	NM_013556.2	CGCAGTCCCAGCGTCGTGATTA	TGAGCACACAGAGGGCCACAA
B2M	*Rattus norvegicus*	NM_012512.2	TCTGCAAGCCTGTGTGCGGT	TGGGGCCAGCACGTCTGAAA
GAPDH	*Rattus norvegicus*	NM_017008.4	TCTTGTGCAGTGCCAGCCTCG	ACCAGGCGTCCGATACGGC
HMBS	*Rattus norvegicus*	NM_013168.2	TTGGAGCCATCTGCAAACGGGAA	CCGTAGGCGGGTGTTGAGGT

Supplementary Table 1: List with primer sequences used for quantitative real-time PCR analyses. Details for all oligonucleotides used in this study are provided.

Supplementary discussion

Blood chemistry analysis from the Su/Hox model revealed none of the severe signs of toxicity often observed in patients on palbociclib, such as neutropenia, thrombocytopenia, and anemia (**Supplementary Figure 9)**. While the number of neutrophils (**Supplementary Figure 9c**) and platelets (**Supplementary Figure 9d**) was reduced in the group of Su/Hox+Palbociclib animals compared to healthy normoxic rats, these values remain within the range of reference values considered normal for this rat strain. We further investigated the right and left ventricle, as well as distinct organs including liver, kidney and intestine, to address issues of cardiotoxicity and gastrointestinal bleeding (**Supplementary Figure 10**). Here, stellate fibrotic lesions in the right ventricle of diseased Su/Hox rats (**Supplementary Figure 10a, arrows**) are particularly noteworthy while there is only a discrete collagen deposition in palbociclib treated Su/Hox animals seen (**Supplementary Figure 10a, arrowhead**). There were no overt signs of inflammation, fibrosis, or necrosis; and the gross presentation of all organs was comparable to that of untreated Su/Hox animals, presenting normotrophic, vital parenchymal cells (**Supplementary Figure 10b**). Indeed, no significant differences were observed between organs from the three experimental groups indicating that the use of palbociclib was safe with regard to blood cell counts and organ damage. CDK isoform expression was also assessed in other organs from healthy rats to assess potential off-target side effects of CDK-targeted therapy. At the protein level, CDK2 (right and left ventricle, thymus), CDK4 (right and left ventricle) and CDK6 (kidney, thymus) were more strongly expressed in other tissues than in the lung (**Supplementary Figure 10c**) but the integrity of those organs was not affected by targeted inhibition by palbociclib.

Supplementary methods

Cell culture of HPAECs

Human pulmonary arterial endothelial cells (HPAECs) from healthy individuals were maintained in culture with endothelial specific growth medium (ECGM) containing 2% FCS, 0.4% endothelial cell growth supplement, 0.1 ng/ml hEGF, 1 ng/ml hFGF-B, 90 µg/ml heparin, 1 µg/ml hydrocortisone (all purchased from PromoCell GmbH, Heidelberg, Germany). All experiments were performed with cells between passages 4 and 6 similar to the HPASMCs and HAoSMCs as stated in the main manuscript. Cells were seeded on different types of dishes according to the different assays. Six hours after seeding, cells were starved for 18 h before subsequent treatments by using endothelial cell basal medium (ECBM; PromoCell) containing only 0.5% FCS but no other supplements. Cells were exposed to inhibitors at concentrations stated in the figures for 24 h in the presence of ECGM.

Upstream kinase prediction

The PamStation platform (PamGene International BV, ´s-Hertogenbosch, Netherlands) allows the prediction of differentially activated kinases present in a biological specimen (e.g. the cell lysate) based on the phosphorylation of their distinct substrates immobilized on the peptide array chip. During the incubation time, active kinases in the lysates phosphorylate their distinct peptide substrates presented on the chip. Phosphorylated peptides are recognized by a phospho-specific FITC-labeled secondary antibody, and detection, performed in multiple cycles at different exposure times, is monitored by a CCD camera. Software-based image analysis integrates the signals obtained within the time course of the incubation of the kinase lysate on the chip into one single value for each peptide for each sample (exposure time scaling). Only peptides that show a steady increase of signal over time on at least a quarter of the arrays are used for further analysis (quality control). In our studies, 100 out of 144 peptides showed a significant alteration in peptide substrate

phosphorylation, which was subsequently used for an upstream kinase analysis. Log transformation of processed signals allows easier graphical presentation of the raw data. Thereby, data with huge differences in intensity are visualized on the log-transformed y-axis in a heat map that shows the degree of phosphorylation for each peptide. In a two-group comparison between both conditions (e.g. healthy and IPAH-HPASMCs), the de-regulation of phosphorylation for each peptide demonstrates the effect of changes in the kinase activity. The analysis of corresponding upstream kinases responsible for selective peptide substrate phosphorylation was performed using Bionavigator (PamGene) software, which allows for kinase identification using post-translational modification (PTM) databases such as HPRD (human protein reference database), PhosphoSitePlus, or databases with in silico predictions such as PhosphoNET, which contains predictions for a relatively large number of kinases. The "code" of (de-)phosphorylation of the different peptides on the chip revealed which kinases are differentially activated between both experimental conditions (e.g. in IPAH-HPASMCs compared with healthy HPASMCs).

Detailed staining protocol for explanted human lungs

As mentioned in the main body, explanted human lungs were fixed in 4% PFA and embedded in paraffin prior their staining according the following procedure. Antibodies specific for CDK2 (#CI996C01) and CDK4 (#CI998C002) were purchased from DCS (Innovative Diagnostik-Systeme, Dr. Christian Sartori GmbH & Co. KG, Hamburg, Germany), while the anti-CDK6 antibody was obtained from Biorbyt Ltd. (#orb135240, Cambridge, UK) and P-Rb from Santa Cruz Biotechnology (#sc-16670-R, Heidelberg, Germany). For the analysis of CDK2 (rabbit anti-CDK2 antibody in 1:50 dilution), CDK4 (mouse anti-CDK4 antibody in 1:50 dilution), CDK6 (rabbit anti-CDK6 antibody in 1:500 dilution), and P-Rb (with a mouse anti-P-Rb antibody in 1:50 dilution) expression, the ZytoChem Plus phosphatase polymer kit (Zytomed Systems GmbH, Berlin, Germany) with the Warp Red Chromogen substrate kit (Zytomed Systems GmbH) was used where positive cells are visible by a purple color. For PCNA detection, FFPE tissue blocks were cooked in rodent decloaker (Biocare

medical by Zytomed Systems GmbH) for antigen retrieval after dehydration (by xylol and ethanol) and incubated in H_2O_2/methanol. After blocking, a mouse anti-PCNA antibody (#2586, Cell Signaling Technology (Danvers, MA, USA) was applied in a 1:50 dilution overnight, followed by development with the ZytoChem Plus peroxidase polymer kit (Zytomed Systems GmbH) with the Nova RED peroxidase substrate (Vector laboratories, Burlingame, CA, USA) leading to a reddish cellular appearance. For those stainings, haematoxylin was used as a counterstain.

For staining of α-SMA (alpha smooth muscle actin) and von-Willebrand factor (vWF), formalin-fixed paraffin-embedded (FFPE) tissue blocks were incubated in xylol to remove paraffin followed by a graded ethanol series to dehydrate the tissue. Blocking of endogenous enzymatic activity was achieved by H_2O_2/methanol. Proteinase K digestion was done prior blocking with bovine serum albumin (BSA) and normal horse serum. Mouse anti-α-SMA antibody (A2547, Sigma-Aldrich now Merck, Darmstadt, Germany) was used in a 1:700 dilution (30 minutes) followed by a biotinylated secondary antibody (30 minutes) and streptavidin-peroxidase complexes provided by the Vectastain universal quick kit (Vector laboratories, Burlingame, CA, USA). Vector® VIP peroxidase substrate from (Vector laboratories, Burlingame, CA, USA) allowed detection as observed by violet staining. After washing and blocking with bovine serum albumin (BSA) and normal horse serum, the tissue was incubated with rabbit anti-vWF antibody (A0082, Dako now Agilent, Hamburg, Germany) was incubated at a final dilution of 1:2000. Detection was carried out with the Quick ImmPress polymer detection kit and the DAB peroxidase substrate kit (both from Vector laboratories, Burlingame, CA, USA) resulting in brownish color. Finally, cells were counterstain with methyl green (Zytomed Systems GmbH) and tissue was mounted after a dehydration process including ethanol, isopropyl alcohol and xylol.

<u>Histology and pathological appraisal of organs from rats</u>

Explanted organs from all animals were fixed in 4% PFA and embedded in paraffin according to standard procedures prior sectioning in 4 µm slices and subsequent staining. FFPE tissue

blocks were incubated in xylol to remove paraffin followed by a graded ethanol series to dehydrate the tissue. Hematoxylin and eosin staining was carried out with acidic hämalaun solution (Waldeck GmbH & Co. KG, Münster, Germany) and eosin Y (Thermo Fisher Scientific, Waltham, MA, USA) as recommended by the manufacturers.

Whole blood analysis from rats

Blood was taken from the right heart ventricle after the animals were anesthetized and their chest was opened prior to assessment of hemodynamic parameters. For whole blood analysis, samples were directly subjected to the flow cytometry-based hematology system (ADVIA 2120, Siemens, Germany) within four hours after blood donation.
