# Supplementary material for: Targeting cyclin-dependent kinases for the treatment of pulmonary arterial hypertension
Source: Nat Commun. 2019 May 17;10:2204. doi: 10.1038/s41467-019-10135-x (PMC6525202; doi:10.1038/s41467-019-10135-x)
Supplement: Supplementary file 3 — Reporting Summary [file 41467_2019_10135_MOESM3_ESM.pdf]

nature research

Corresponding author(s): Ralph Schermuly (NCOMMS-18-10990B)

Last updated by author(s): Feb 12, 2019

Reporting Summary

Nature Research wishes to improve the reproducibility of the work that we publish. This form provides structure for consistency and transparency in reporting. For further information on Nature Research policies, see Authors & Referees and the Editorial Policy Checklist.

Statistics

For all statistical analyses, confirm that the following items are present in the figure legend, table legend, main text, or Methods section.

n/a	Confirmed	
☐	☒	The exact sample size (*n*) for each experimental group/condition, given as a discrete number and unit of measurement
☐	☒	A statement on whether measurements were taken from distinct samples or whether the same sample was measured repeatedly
☐	☒	The statistical test(s) used AND whether they are one- or two-sided<br>*Only common tests should be described solely by name; describe more complex techniques in the Methods section.*
☐	☒	A description of all covariates tested
☐	☒	A description of any assumptions or corrections, such as tests of normality and adjustment for multiple comparisons
☐	☒	A full description of the statistical parameters including central tendency (e.g. means) or other basic estimates (e.g. regression coefficient) AND variation (e.g. standard deviation) or associated estimates of uncertainty (e.g. confidence intervals)
☐	☒	For null hypothesis testing, the test statistic (e.g. *F*, *t*, *r*) with confidence intervals, effect sizes, degrees of freedom and *P* value noted<br>*Give P values as exact values whenever suitable.*
☒	☐	For Bayesian analysis, information on the choice of priors and Markov chain Monte Carlo settings
☒	☐	For hierarchical and complex designs, identification of the appropriate level for tests and full reporting of outcomes
☒	☐	Estimates of effect sizes (e.g. Cohen's *d*, Pearson's *r*), indicating how they were calculated

Our web collection on statistics for biologists contains articles on many of the points above.

Software and code

Policy information about availability of computer code

Data collection	The PamStation®12 platform (PamGene International, s-Hertogenbosch, Netherlands) with Evolve 12 software (2nd release 0.08) was used for data collection in the peptide-based kinase activity assay (https://www.pamgene.com/en/pamstation.htm)
Data analysis	Bionavigator software v. 6.2 (PamGene) was used to predict upstream kinase activity based on peptide substrate phoshorylation. https://www.pamgene.com/en/bionavigator.htm

For manuscripts utilizing custom algorithms or software that are central to the research but not yet described in published literature, software must be made available to editors/reviewers. We strongly encourage code deposition in a community repository (e.g. GitHub). See the Nature Research guidelines for submitting code & software for further information.

Data

Policy information about availability of data

All manuscripts must include a data availability statement. This statement should provide the following information, where applicable:

- Accession codes, unique identifiers, or web links for publicly available datasets
- A list of figures that have associated raw data
- A description of any restrictions on data availability

The data that support the findings of this study are available from the corresponding author upon reasonable request.

Field-specific reporting

Please select the one below that is the best fit for your research. If you are not sure, read the appropriate sections before making your selection.

☒ Life sciences ☐ Behavioural & social sciences ☐ Ecological, evolutionary & environmental sciences

Life sciences study design

All studies must disclose on these points even when the disclosure is negative.

Sample size	Sample sizes were determined depending on the experimental settings and availability of the biospecimen. Due to limitations in the availability and the amount of healthy human lung tissue and subsequently isolated primary cells (healthy HPASMCs), we have lower n-numbers as in the case of IPAH diseased biospecimen (and IPAH-HPASMCs). For in vivo experiments, we were limited to certain sample sizes due to the animal proposal. All of our findings were only reported to be conclusive and successfull if statistical significance was obtained. All n-numbers and the type of replicates (i.e. biological versus technical) are mentioned in the corresponding figure legend and can be found in the soure data file.
Data exclusions	Data points were excluded in few cases if they could be identified as outliers due to technical failures during the experimental process. This indicated in the source data file.
Replication	All our findings were clearly reproducible as indicated by using multiple biological as well as technical replicates and independently repeated measurements.
Randomization	For the in vivo study, diseased animals (MCT- or Su5416-injected) were randomly allocated to the two groups i.e. placebo (MCT ; Su/Hox) or treatment (MCT + Palbociclib; Su/Hox + Palbociclib).
Blinding	In vitro studies were not performed in a blinded fashion due to the high risk of confusion in handling of the samples by different experimenters. For the quantification of IHC stainings and pathological evaluation of animal speicmen, the investigator were blinded to ensure an unbiased interpretation of the results.

Reporting for specific materials, systems and methods

We require information from authors about some types of materials, experimental systems and methods used in many studies. Here, indicate whether each material, system or method listed is relevant to your study. If you are not sure if a list item applies to your research, read the appropriate section before selecting a response.

Materials & experimental systems

n/a	Involved in the study	
☐	☒	Antibodies
☐	☒	Eukaryotic cell lines
☒	☐	Palaeontology
☐	☒	Animals and other organisms
☒	☐	Human research participants
☒	☐	Clinical data

Methods

n/a	Involved in the study	
☒	☐	ChIP-seq
☐	☒	Flow cytometry
☒	☐	MRI-based neuroimaging

Antibodies

Antibodies used

Antibodies for Western blot analysis:
- Cleaved Caspase-3, #9665, Cell Signaling Technology (Danvers, MA, USA)
- P-CDK2, #2561, Cell Signaling Technology (Danvers, MA, USA)
- CDK2, #2546, Cell Signaling Technology (Danvers, MA, USA)
- CDK4, #12790, Cell Signaling Technology (Danvers, MA, USA)
- P-CDK6, #ab131439, Abcam (Cambridge, UK)
- CDK6, #3136, Cell Signaling Technology (Danvers, MA, USA)
- P-CDK9, #2549S, Cell Signaling Technology (Danvers, MA, USA)
- CDK9, #2316S, Cell Signaling Technology (Danvers, MA, USA)
- Cyclin D1, #2978, Cell Signaling Technology (Danvers, MA, USA)
- Cyclin D3, #2936, Cell Signaling Technology (Danvers, MA, USA)
- PCNA, #sc-56, Santa Cruz Biotechnology (Heidelberg, Germany)
- P-ERK1/2, #4370, Cell Signaling Technology (Danvers, MA, USA)
- ERK1, #sc-93, Santa Cruz Biotechnology (Heidelberg, Germany)
- P-Rb, #8516S, Cell Signaling Technology (Danvers, MA, USA)
- Rb, #9309S, Cell Signaling Technology (Danvers, MA, USA)
- Beta-actin, #ab8226, #ab8227, Abcam (Cambridge, UK)
- GAPDH, #2118, Cell Signaling Technology (Danvers, MA, USA)
- Vinculin, #ab18058, Abcam (Cambridge, UK)
- anti-rabbit/mouse HRP-labeled secondary antibody, #7074S/#7076S, (Cell Signaling Technology)

Antibodies for immune histochemistry:
- CDK2, #CI996C01, DCS (Innovative Diagnostik-Systeme, Hamburg, Germany)
- CDK4, #CI998C002, DCS (Innovative Diagnostik-Systeme, Hamburg, Germany)
- CDK6, #orb135240, Biorbyt Ltd. (Cambridge, UK)

	- PCNA, #2586, Cell Signaling Technology (Danvers, MA, USA)<br>- P-Rb, #sc-16670-R, Santa Cruz Biotechnology (Heidelberg, Germany)<br>- α-SMA, #A2547, Sigma-Aldrich now Merck (Darmstadt, Germany)<br>- vWF, #A0082, Dako now Agilent (Hamburg, Germany)
Validation	All antibodies were carefully selected for the desired application and utilized according to the manufacturer´s instructions. Validation statements can be found on the suppliers website and related positive/negative controls were performed in parallel in our study. Unfortunately, not all antibodies (claimed to be specific) for the various CDK isoforms can discriminate between those homologous proteins and their phosphorylation in all applications (i.e. WB and IHC) in humans and rats. We also tried several additional protocols to establish IHC stainings for P-CDK2 and P-CDK6, but none of these antibodies showed reliable results. Especially on specimen from our laboratory rats, IHC analyses did not lead to a trustworthy outcome. In total, we tested multiple antibodies for all the proteins from the following companies: Abcam, Biorbyt, Cell signaling, DCS (Innovative Diagnostik-Systeme), R&D systems, and Santa Cruz Biotechnology. In our study we used the best working ones giving a specific signal; those are listed above (see section antibodies used).

Eukaryotic cell lines

Policy information about cell lines

Cell line source(s)	Only isolated primary cells were used which were provided from Lonza Ltd. (Basel, Switzerland) as well as from the UGMLC Giessen Biobank.<br>- Human pulmonary arterial smooth muscle cells (HPASMCs)<br>- Human aortic smooth muscle cells (HAoSMCs)<br>- Human pulmonary arterial endothelial cells (HPAECs)
Authentication	Cells obtained from Lonza Ltd. (Basel, Switzerland) were authenticated by the manufacturer (e.g. by expression of alpha actin). Cells provided by the UGMLC Giessen Biobank were characterized by immune fluorescence staining positive for SMA and negative for vWF or vimentin.
Mycoplasma contamination	All cells were tested negative for mycoplasma contamination by PCR prior to our studies.
Commonly misidentified lines<br>(See ICLAC register)	*Name any commonly misidentified cell lines used in the study and provide a rationale for their use.*

Animals and other organisms

Policy information about studies involving animals; ARRIVE guidelines recommended for reporting animal research

Laboratory animals	MCT model: Sprague-Dawley rats, male, 300-350 g body weight<br>Su/Hox model: Wistar-Kyoto rats, male, 300-350 g body weight<br>Body weight was chosen as specific parameter for proper dose calculation and drug administration.
Wild animals	The study did not involve wild animals.
Field-collected samples	The study did not involve samples collected from the field.
Ethics oversight	Both, the University Animal Care Committee and the federal authorities for animal research of the Regierungspräsidium Giessen and Darmstadt (Hesse, Germany) approved the study protocol (approval numbers V 54 - 19 c 20 15 h 01 GI 20/10 Nr. G 50/2016 and V54-19c 20/15-B2/1195).

Note that full information on the approval of the study protocol must also be provided in the manuscript.

Flow Cytometry

Plots

Confirm that:

☒ The axis labels state the marker and fluorochrome used (e.g. CD4-FITC).

☒ The axis scales are clearly visible. Include numbers along axes only for bottom left plot of group (a 'group' is an analysis of identical markers).

☒ All plots are contour plots with outliers or pseudocolor plots.

☒ A numerical value for number of cells or percentage (with statistics) is provided.

Methodology

Sample preparation	Cell cycle analysis: Cells were cultured in 10-cm dishes and starved for 18 h in BM. Treatment with CDK inhibitors was performed in the presence of GM-2 for 24 h. Cells and conditioned media were collected by trysinization followed by centrifugation at 600 g for 5 min at 4°C with brakes disabled; pellets were washed twice in ice-cold PBS and fixed overnight in 70% ethanol at 4°C. Thereafter, cells were washed in 38 mM sodium citrate buffer (pH 7.4) and incubated in 300 µl hypotonic DNA staining solution (0.05 mg/ml PI, 5 µg/ml RNase A and 38 nM sodium citrate) at room temperature for 30 min protected from light. Flow cytometric measurement was carried out with a BD FACSCANTO II flow cytometer. Analysis of the different cell cycle phases

(Sub-G1, G1, S and G2/M) was performed with BD FACSDiva Software (Version 6.1.3), and mean values from four individual sets of experiments were recorded. A figure exemplifying the gating strategy is provided in the supplementary information (Supplementary Figure 12 a-c).
Apoptosis detection assay: Detailed analysis of apoptosis-mediated cell death was performed by classical annexin V (AV) staining combined with propidium iodid (PI) using the FITC Annexin V Apoptosis Detection Kit I (BD Bioscience, Heidelberg, Germany) according to the manufacturer's guidelines. In summary, as in the conditions used for the cell cycle analyses, HPASMCs or HAoSMCs were seeded in triplicate, starved, and treated for 24 h with the different compounds. For sample preparation, cells including cell culture supernatant were collected by accutase digestion (PAN-Biotech GmbH, Aidenbach, Germany), centrifuged at 600 g for 5 min at 4°C with brakes disabled, and carefully washed twice with cold PBS. Cell pellets were resuspended in 200 µl AV binding buffer to a concentration of 106 cells per ml, from which 100 µl were stained with 5 µl of FITC-conjugated AV-FITC and 5 µl PI solution. Cells were incubated for 15 min at room temperature in the dark and further diluted with 100 µl AV binding buffer prior to being assessed within 1 h by flow cytometry with detection of PI in the PerCP-Cy5.5 channel. A figure exemplifying the gating strategy is provided in the supplementary information (Supplementary Figure 12 d-f). Data from three independent experiments performed in triplicate were analyzed by FlowJo software (Version 10) and are summarized in one graph.

Instrument	BD FACSCANTO II flow cytometer
Software	Cell cycle analysis: BD FACSDiva software (Version 6.1.3)<br>Apoptosis detection assay: FlowJo software (Version 10)
Cell population abundance	For both analysis, all cells were analyzed for their DNA content (cell cycle analysis) or the presence of apoptotic markers (AV/PI detection). Neither sorting nor enrichment was conducted for these isolated primary HPASMCs and HAoSMCs.
Gating strategy	Cell cycle analysis: Cells were first assessed in the FSC-A/SSC-A dot plot to exclude cell debris (P1) and doublet discrimination was carried out by additional plots to remove doublets and cell clumps (P2 and P3). Finally, fragmented DNA (low signals) as well as the different cell cycle phases (increasing signals) were recorded. Linear (orange, PE-A) and logarithmic (purple, PerCP-A) scaling of the x-axis led to identical event counts for the SubG1 population. An unstained as well as a positive control sample was used to define PI-positive cells and to identify fragmented DNA. A detailed description of the experimental design can be found in the main manuscript or in the supplementary material.<br>Apoptosis detection assay: For apoptosis detection, samples were analysed by FSC-A/SSC-A dot plot and cell debris was removed (P1). Next, doublets were excluded (P2) and finally, fluorescence was detected in the FITC (AV) and PerCP-Cy5.5 (PI) channel. Proper positive and negative as well as unstained and single-stained control samples were used to define the four different populations, to apply quadrant statistics and to exclude debris. Tools for fluorescence compensation were applied whenever needed. A detailed description of the experimental design can be found in the main manuscript or in the supplementary material.

☒ Tick this box to confirm that a figure exemplifying the gating strategy is provided in the Supplementary Information.
